# Graph attention and text semantics improve personalized recommendation

**DOI:** 10.1038/s41598-026-46737-x

**Published:** 2026-04-07

**Authors:** Jing Dong, Ziyu Shen, Hao Luo, Tianyi Lyu, Weixuan Gao

**Affiliations:** 1 Fu Foundation School of Engineering and Applied Science, Columbia University, New York, NY 10027 USA; 2 Department of Computer Science, Rutgers University, Santa Clara, CA 94301 USA; 3 Department of Electrical & Computer Engineering, Northeastern University, Quincy, MA 02171 USA; 4 Department of Civil and Environmental Engineering, Stanford University, Palo Alto, CA 94305 USA

**Keywords:** Engineering, Mathematics and computing

## Abstract

With the increasing challenge of information overload, personalized recommendation systems play an essential role in delivering relevant content to users. Traditional collaborative filtering methods often suffer from data sparsity and cold-start problems, which limit their effectiveness in real-world applications. To address these issues, this paper proposes a personalized recommendation model that integrates graph attention mechanisms with auxiliary textual information. User–item interactions are modeled on a knowledge graph, where graph attention networks are employed to capture multi-hop user interests, while graph convolution is used to aggregate item-side neighborhood information. Textual data associated with users and items are encoded into semantic embeddings and incorporated to enrich the initial representations of graph entities. Experiments conducted on the Book-Crossing and MovieLens-1M datasets demonstrate that the proposed model achieves superior performance in terms of AUC, F1-score, and Top-K recall compared with several state-of-the-art baselines. The results indicate that combining graph-based modeling with textual semantic enhancement can effectively improve recommendation accuracy and robustness under sparse data conditions.

## Introduction

In recent years, the rapid growth of the internet and the surge of information have brought significant attention to recommendation systems as a solution to ”information overload”^1,2^. The main function of these systems is to predict users’ potential interest in new items by analyzing their past behaviors and preferences. Collaborative Filtering (CF)^3^, a widely used recommendation algorithm, operates by measuring the similarity between users or items for generating recommendations^4,5^. However, CF’s heavy reliance on the user-item rating matrix causes its performance to decline sharply in the presence of sparse data, prompting extensive research aimed at addressing this issue.

To address the sparsity of the rating matrix, many researchers have incorporated text features into recommendation systems, particularly by analyzing users’ historical reviews to complement the lack of rating information. Text data not only provide rating information but also reveal users’ fine-grained preferences and emotional tendencies. By mining user reviews, recommendation systems can better understand user needs and improve recommendation accuracy. For example, models such as DeepCoNN^6,7^ and NARRE^8^ utilize neural networks to extract features from user reviews and item descriptions, then integrate these features with collaborative filtering to partially address the data sparsity issue.

While introducing text features into recommendation systems has enhanced recommendation performance, most existing approaches treat all review texts equally, ignoring the dynamic interaction between users and items. Additionally, these methods typically extract static text features, limiting their ability to capture complex user-item interactions effectively. Therefore, a key challenge remains in how to better leverage fine-grained interactions within review texts in recommendation algorithms^9–11^. Knowledge graphs^12,13^, as a structured form of semantic representation, have also gained attention in recommendation systems. Knowledge graphs can supplement explicit user-item relationships and mine potential preference information through semantic associations. Knowledge graph-based recommendation approaches mainly consist of embedding methods, path-based methods, and propagation methods^14^. Embedding methods model entities and relations within the graph by embedding them into a low-dimensional space; path-based methods identify user-item connections through graph paths; and propagation methods capture high-order interactions through information diffusion^15^. These approaches have shown strong performance in semantically rich contexts, effectively improving recommendation accuracy. However, they also present challenges, such as introducing noise through excessive interactions and high computational costs in sparse datasets. Thus, a critical direction in recommendation research is finding ways to efficiently model user-item interactions while minimizing noise from graph structures.

**Fig. 1:**
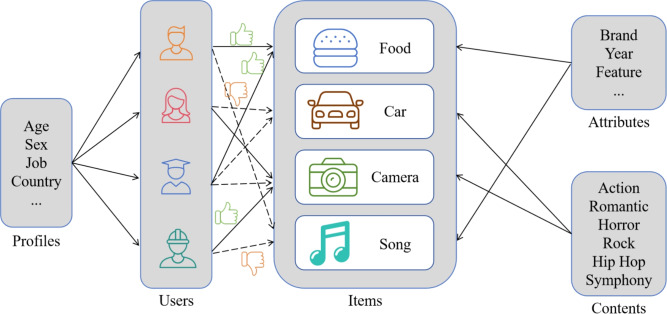
Logical process of recommendation system.

Graph Neural Networks (GNNs)^16^ have demonstrated powerful capabilities in handling graph-structured data. A variant, Graph Attention Networks (GATs)^17^, assigns different weights to nodes and edges, dynamically capturing complex relationships between nodes. Incorporating GAT into recommendation systems can help capture intricate user-item interactions, especially under sparse data conditions. For example, the TKGAT^18^ model proposed by Wang et al. leverages GAT to aggregate neighboring information from multiple iterative sets, yielding strong recommendation performance. However, there are still limitations in capturing dynamic user preferences, particularly in modeling changes over time.

In personalized recommendation systems^?^, deep learning models have evolved to address challenges such as data sparsity, recommendation accuracy, and identifying user preferences. Matrix factorization^19,20^, a traditional CF method, breaks down the user-item rating matrix into two low-dimensional matrices that capture latent features of users and items to predict ratings. While it handles sparse matrices well, it only relies on rating data, failing to capture complex relationships and dynamic user interests. Deep collaborative filtering combines matrix factorization with neural networks^21^, using non-linear mappings to capture more intricate user-item relationships. Attention mechanisms^22^, which capture global dependencies in user behaviors, are particularly suitable for handling complex user behavior sequences.

Therefore, combining graph attention mechanisms with big data text features in personalized recommendation algorithms is highly significant. First, GATs can dynamically model user-item interactions, enhancing recommendation accuracy and personalization. Second, text features enrich the input to recommendation systems, addressing the traditional problem of sparse rating matrices. Integrating these two aspects can substantially improve the performance of recommendation systems, better addressing the diverse needs of users and the sparsity of data. Figure 1 outlines the logical process of modern recommendation systems.

This paper proposes a novel personalized recommendation algorithm that integrates GATs with big data text features to overcome the limitations of existing recommendation systems in sparse datasets and text feature extraction. Experimental results demonstrate that this approach significantly improves recommendation accuracy, personalization, and system robustness, providing new insights for future research in recommendation systems.

## Related work

In personalized recommendation systems, the rapid expansion of data and the growing complexity of user preferences have highlighted the shortcomings of traditional algorithms like Collaborative Filtering (CF) and matrix factorization, especially regarding data sparsity and cold-start issues. To overcome these challenges, researchers have begun incorporating knowledge graphs, Graph Neural Networks (GNN), and large-scale text features into recommendation systems, which has led to significant improvements in both accuracy and resilience.

Recently, Graph Attention Networks (GATs) have gained traction in this domain. By assigning varying weights to nodes and edges, GATs effectively capture intricate relationships between users and items. For instance, Deng et al. introduced the GAKG model^23^, which integrates knowledge graph embeddings with attention aggregation networks, resulting in enhanced performance in personalized recommendation tasks. This model leverages users’ historical interactions and employs GNNs to model knowledge graph data, helping to mitigate data sparsity challenges. Similarly, Sun et al. demonstrated the benefits of multimodal knowledge graphs, particularly in areas like music, book, and movie recommendations.

Beyond recommendation systems, knowledge graphs have also been successfully applied in other domains to organize complex semantic information and support downstream learning tasks. Guo et al.^24^ proposed a knowledge graph–aided framework for crowdsourced requirements generation in software testing, where structured graph representations are used to capture domain knowledge and guide automatic requirement construction. Although this work focuses on software testing rather than recommendation, it highlights the effectiveness of knowledge graphs in modeling heterogeneous entities and relationships, which is conceptually aligned with graph-based representation learning in recommender systems.

Recent studies on multi-hop knowledge graph reasoning further demonstrate the importance of contextual information aggregation. Zheng et al.^25^ introduced a context-aware adaptive reinforcement learning framework for multi-hop reasoning in few-shot knowledge graph scenarios. Their work emphasizes dynamic context modeling and multi-hop path exploration, which is closely related to the multi-hop propagation mechanisms adopted in graph-based recommendation models. While their method targets knowledge graph completion rather than recommendation, it provides valuable insights into controlling information propagation and leveraging contextual dependencies in multi-hop graph reasoning.

The rise of big data has further propelled the development of personalized recommendation algorithms. Modern systems leverage big data processing frameworks, such as Hadoop and Spark, to efficiently handle vast amounts of unstructured data. Research shows that big data platforms allow parallel processing of diverse data sources, making recommendation computation more efficient and capable of handling large datasets and varied user behaviors. Additionally, these technologies enable finer-grained modeling of user behaviors and item attributes, improving both recommendation accuracy and responsiveness.

Among systems combining GNNs and text features, the KGAT (Knowledge Graph Attention Network)^26^ model stands out as a key example. KGAT builds user-item knowledge graph relationships and applies attention mechanisms to address cold-start and data sparsity issues in recommendation tasks. Graph Convolutional Networks (GCNs)^27^ are also widely adopted in large-scale recommendation systems, efficiently capturing high-order relationships between users and items. For instance, Wang et al. introduced the Neural Graph Collaborative Filtering model, which applies graph-based collaborative filtering to achieve more effective personalized recommendations.

In summary, the evolution of personalized recommendation systems is moving towards deep learning, multimodal fusion, and knowledge graph embedding. Knowledge graph embeddings enrich recommendation systems with semantic information, while attention mechanisms help systems focus on key user behavior patterns. Future research is likely to explore how to combine big data text features, knowledge graphs, and GNNs more effectively to further enhance the performance of personalized recommendation systems.

## Methods

**Fig. 2:**
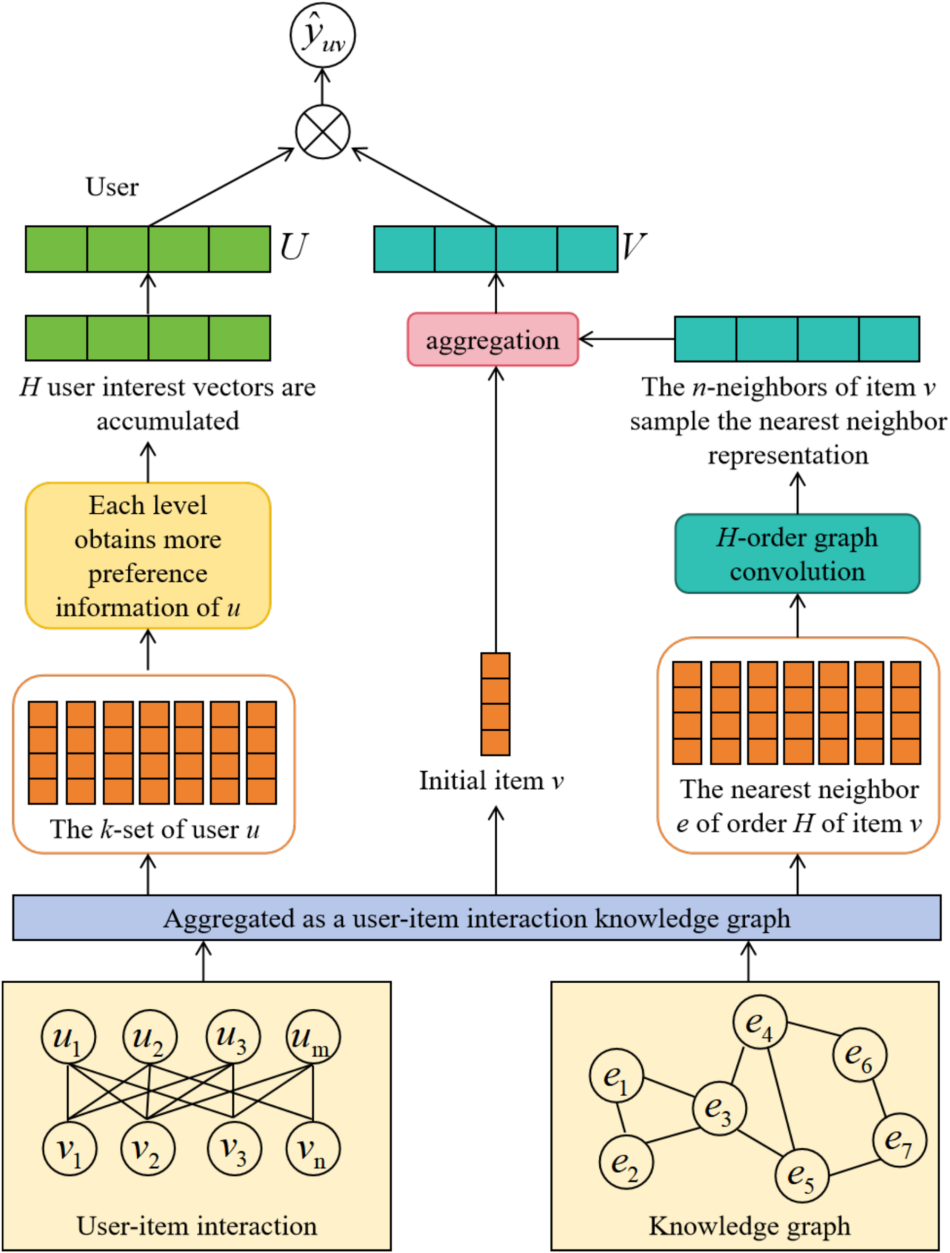
TNRA model overall framework.

This paper presents a personalized recommendation algorithm that combines a graph attention mechanism with large-scale text features to enhance performance in sparse data scenarios. The method uses user interaction history and text reviews, recommending items by constructing a knowledge graph. The TNRA model framework is illustrated in Fig. 2. Specifically, let the user set be defined as $U=\{u_{1},u_{2},...,u_{m}\}$, and the item set be $V=\{v_{1},v_{2},...,v_{n}\}$. The user-item interaction matrix $Y \in \mathbb {R}^{m\times n}$ is defined based on users’ implicit feedback, where $y_{uv}=1$ indicates interaction between user *u* and item *v* (such as clicking or browsing), and $y_{uv}=0$ otherwise. We denote the *d*-dimensional embedding vectors of user *u* and item *v* as $u\in \mathbb {R}^{d}$ and $v\in \mathbb {R}^{d}$, respectively. The algorithm introduces a knowledge graph *G*, composed of triples (*h*, *r*, *t*), where $h,t \in E$ are head/tail entities and $r \in R$ is the relation type. *E* and *R* are the entity and relationship sets in the knowledge graph. Each entity $e\in E$ and relation $r\in R$ is associated with an embedding vector $e\in \mathbb {R}^{d}$ and $r\in \mathbb {R}^{d}$.

First, on the user side, the potential interest set $E^k_u$ is constructed for each user by traversing multi-hop ripple sets. A single attention coefficient $\alpha$ is used to weight different triples when aggregating multi-hop neighbors, producing the *k*-hop user interest representation $M_u^{k}$. Second, on the item side, GCNs and neighbor sampling are used to obtain the item neighborhood topology. By aggregating the user-preference-weighted neighbors, the final item vector representation $v_{N_v}^{u}$ is formed. Finally, the user and item aggregation vectors *U* and *V* are multiplied to compute the probability $\hat{y}_{uv}=\sigma (U^{T}V)$ that user *u* will click item *v*. To prevent overfitting, a label smoothing regularization term is introduced, and cross-entropy loss $L_{loss}$ is used for optimization. The proposed method demonstrates significant improvements in recommendation accuracy and system robustness through experiments.

### Text feature encoding and integration

In order to alleviate the sparsity of user–item interactions, textual information associated with users and items is utilized as auxiliary semantic features in our model. The text data mainly consist of user-generated reviews and item-related textual descriptions, which provide complementary preference signals beyond implicit feedback.

All textual data are first preprocessed through tokenization and noise removal. Each word is mapped into a low-dimensional continuous vector using pre-trained word embeddings. To obtain fixed-length text representations, a convolutional neural network (CNN) followed by a max-pooling operation is applied to capture local semantic patterns from the text. The resulting vectors represent the semantic features of users or items derived from textual information.

Rather than modeling text as a separate prediction branch, the extracted text embeddings are fused with the corresponding user or item embeddings in the knowledge graph. Specifically, for each entity, its graph-based embedding is concatenated with the associated text embedding to form an enhanced initial representation. These fused embeddings are then used as input node features for the subsequent graph attention aggregation on the user side and graph convolution aggregation on the item side.

By integrating text features at the embedding level, the proposed method enriches semantic representations while maintaining compatibility with the graph-based propagation and attention mechanisms.

### User-side neighbor information aggregation

**Fig. 3:**
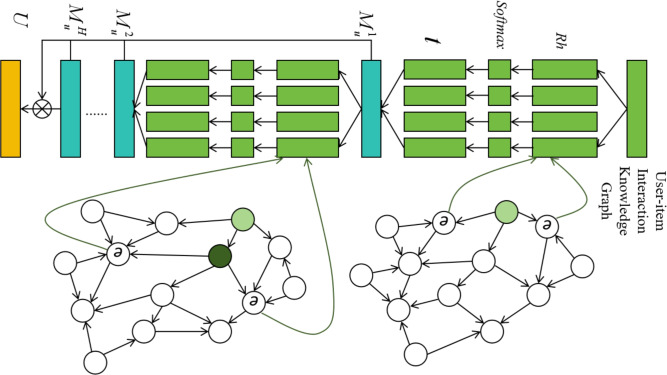
User-side neighbor information aggregation process.

Figure 3 illustrates the user-side neighbor aggregation process, where this method integrates GAT to dynamically capture users’ historical interests and potential preferences. The fundamental idea behind this approach is to traverse the user’s historical interaction data, identifying entities related to the user in the knowledge graph, and propagating the user’s interests layer by layer through multi-hop ripple sets. This aggregation approach efficiently leverages implicit user feedback, capturing potential associations between users and items to enhance recommendation accuracy.

Specifically, let the user set be $U=\{u_{1},u_{2},...,u_{m}\}$ and the item set be $V=\{v_{1},v_{2},...,v_{n}\}$. The interaction between users and items is defined by the implicit feedback matrix $Y\in \mathbb {R}^{m\times n}$, where $y_{uv}=1$ indicates that user *u* has interacted with item *v* (such as clicking or browsing), and $y_{uv}=0$ indicates no interaction. On the user side, we start from the user’s historical interacted items and construct multi-hop ripple sets on the knowledge graph. For each triple (*h*, *r*, *t*), we denote the head and tail entity embeddings as $e_{h}\in \mathbb {R}^{d}$ and $e_{t}\in \mathbb {R}^{d}$, and the relation embedding as $r\in \mathbb {R}^{d}$. For a target item *v*, its embedding is denoted as $v\in \mathbb {R}^{d}$.

First, we define the user *u*’s set of neighboring entities at the *k*-th hop in the graph as:

1 $$\begin{aligned} E_{u}^{k}=\{t|(h,r,t)\in G,h\in E_{u}^{k-1}\},k=1,2,...,H, \end{aligned}$$

Where *G* is the knowledge graph, *H* denotes the maximum number of hops, and $E_u^k$ represents the set of neighboring entities at the *k*-th hop. During each hop, the user’s interest expands from their historical interactions and propagates through the links in the knowledge graph. Next, we define the ripple set at the *k*-th hop for user *u* as:

2 $$\begin{aligned} S_{u}^{k}=\{(h,r,t)\mid (h,r,t)\in G,\,h\in E_{u}^{k-1}\},\;k=1,2,...,H, \end{aligned}$$

To compute user *u*’s preference-aware aggregation for a candidate item *v*, we assign a single attention coefficient $\alpha$ to each triple in the ripple set. For hop *k*, for each triple $(h,r,t)\in S_u^{k}$, we compute an unnormalized attention score as:

3 $$\begin{aligned} p_{j}=\textrm{softmax}\left( v^{\textrm{T}}R_{j}h_{j}\right) =\frac{\exp \left( v^{\textrm{T}}R_{j}h_{j}\right) }{\sum _{(h,r,t)\in S_{u}^{1}}\exp \left( v^{\textrm{T}}Rh\right) }, \end{aligned}$$

Where $R_{j}$ is the linear transformation matrix, and $h_j$ and *v* represent the historical preferences of the user and the item feature vector, respectively. Next, we compute the relationship weight $q_i$ for the neighboring nodes using an attention mechanism:

4 $$\begin{aligned} q_{j}=\frac{\exp {(A(e_{h_{j}},r_{j},e_{t_{j}}))}}{\sum _{(h,r,t)\in S_{u}^{1}}\exp {(A(e_{h},r,e_{t}))}}, \end{aligned}$$

Where $A(e_{h},r,e_{t})$ is a scoring function reflecting the importance of the triple *h*, *r*, *t*. Then, the final relationship weight $n_j$ is obtained by aggregating the relevance scores and attention weights:

5 $$\begin{aligned} n_{j}=\textrm{softmax}(p_{j}+q_{j}), \end{aligned}$$

By multiplying the relationship weights with the tail entities in the aggregated message, we obtain the user *u*’s interest representation for item *v*:

6 $$\begin{aligned} M_{u}^{1}=\sum _{(h_{f},r_{f},t_{f})\in S_{u}^{1}}n_{j}\boldsymbol{t}_{j}, \end{aligned}$$

After multiple hops of diffusion, the user’s interest vectors $M_{u}^{2},M_{u}^{3},...,M_{u}^{H}$ are sequentially generated. The final aggregated user vector is represented as:

7 $$\begin{aligned} U=M_{u}^{1}+M_{u}^{2}+\cdots +M_{u}^{H}, \end{aligned}$$

This multi-hop diffusion and graph attention mechanism allow the model to capture user preferences and potential interests more granularly, leading to more accurate recommendations.

### Item-side neighbor information aggregation

In the item-side neighbor aggregation part, this method uses GCNs to aggregate the neighborhood information of items and combines the GAT to dynamically adjust the weights of neighboring nodes, generating more precise item feature representations. The primary function of this part is to capture the potential relationships between items through the transmission of information between neighbors and, combined with user preference weights, achieve more personalized recommendations.

First, we define the item set as $V=\{v_{1},v_{2},...,v_{n}\}$, and the relationships between items are represented by the knowledge graph *G*, where a triple $S(h,r,\bar{t})$ represents the relationship between item and its neighboring node. We aggregate the neighbors of item *v* using graph convolution operations. The neighborhood topology of item can be expressed as follows:

8 $$\begin{aligned} N_{v}=\{e|(v,r,e)\in G\}, \end{aligned}$$

Where $N_v$ is the neighborhood set of item *v*, representing the connections between item *v* and its neighboring entities through relationship *r*.

To capture the user’s preference for the item’s neighbors, we introduce a preference weight $\psi _{r}^{u}$ for each relationship *r*, which is calculated as follows:

9 $$\begin{aligned} \psi _{r}^{u}=a(u,r), \end{aligned}$$

Where *a*(*u*, *r*) is the dimensionality-reduced inner product between the user vector *u* and the relationship vector *r*, representing the user ’s preference for relationship *r*. Based on the user’s preference weight, the relationship weight $\widetilde{\psi }_{r_{v,e}}^{u}$ between the neighbor node *e* and item *v* is calculated using the normalization formula:

10 $$\begin{aligned} \widetilde{\psi }_{r_{v,e}}^{u}=\frac{\exp \left( \psi _{r_{v,e}}^{u}\right) }{\sum _{e\in N_{v}}\exp \left( \psi _{r_{v,e}}^{u}\right) }, \end{aligned}$$

Where $\psi _{rv,e}^{u}$ represents the user’s preference weight for the relationship between item *v* and its neighboring entity *e*.

Next, to obtain the final aggregated representation of the neighborhood for item *v*, the information of its neighbors is aggregated by weighted summation as follows:

11 $$\begin{aligned} v_{N_{v}}^{u}=\sum _{e\in N_{v}}\widetilde{\psi }_{r_{v,s}}^{u}\cdot e, \end{aligned}$$

Where $v_{N_{\nu }}^{u}$ is the weighted aggregation result of the neighbor information, representing the neighborhood information of item *v*.

Considering that the scale of an item’s neighborhood can be large, if not controlled, it may expand to nodes far from the target item, introducing unnecessary noise. Therefore, we adopt a random sampling technique to sample a fixed number of neighbor nodes from the neighborhood set $N_v$:

12 $$\begin{aligned} T_{v}=\{e|e\sim N_{v},|T_{v}|=N\}, \end{aligned}$$

Where *N* is a constant representing the number of sampled neighbors. Through this method, the model can effectively control the scale of neighbor nodes, avoiding the spread of noise.

Next, the aggregated neighbor vector $v_{N_{\nu }}^{u}$ is combined with the item’s own feature vector *v*, using a bi-interaction aggregation method, which includes vector addition and element-wise product operations, as shown below:

13 $$\begin{aligned} \begin{gathered} agg_{bi-Interaction}(v,v_{T_{v}}^{u})\\ =\textrm{LeakyReLU}\left( W_{1}\left( v+v_{T_{v}}^{u}\right) \right) \\ +\textrm{LeakyReLU}(W_{2}(v\odot v_{T_{v}}^{u}))\end{gathered}, \end{aligned}$$

Where $W_1$ and $W_2$ are linear transformation matrices, and $\odot$ represents the element-wise product operation. By this means, we can effectively combine the neighborhood information of the item with its own information, generating the final vector representation *v* of the item:

14 $$\begin{aligned} V=\sigma (W_{3}\cdot \arg _{\mathrm {bi-Interaction}}(v,v_{T_{v}}^{u})+b), \end{aligned}$$

Where $W_3$ is the linear transformation matrix, $\sigma$ is the non-linear activation function, and *b* is the bias term.

Through this neighborhood information aggregation process, the model can more precisely capture the neighborhood feature information of items, and combined with the user’s preferences, enhance the personalization and accuracy of the recommendation results.

### Probability prediction

In the probability prediction phase, this method calculates the probability of a user clicking on an item by combining the aggregated vectors from the user side and the item side, thereby generating personalized recommendation results. The core task of this phase is to use the aggregated user vector *U* and the aggregated item vector *V* to compute the probability that a user will click on an item using the dot product, while introducing a loss function to optimize the model’s predictive ability.

First, the interest representation vector *U* for user *u* and the aggregated vector *V* for item *v* are obtained from the previous two sections. The probability that user *u* clicks on item *v*, denoted as $\hat{y}_{uv}$, is calculated by taking the dot product of these two vectors:

15 $$\begin{aligned} \hat{y}_{uv}=\sigma (U^{\textrm{T}}V), \end{aligned}$$

Where $\sigma (x)=\frac{1}{1+e^{-x}}$ is the sigmoid function, which maps the result of the dot product to the range (0, 1), representing the click probability.

To improve the model’s generalization ability and prevent overfitting, the model introduces a label smoothing regularization term $L_{ls}$. Label smoothing assumes that items with similar interests in the knowledge graph should have similar labels, meaning neighboring items may share similar user preference scores. The label smoothing is calculated using the following formula:

16 $$\begin{aligned} \hat{y}_{UV}^{\textrm{LS}}=\frac{1}{|N_{v}|}\sum _{e\in N_{v}}\tilde{\psi }_{r_{v,e}}^{u}y_{ue}, \end{aligned}$$

Where $N_v$ represents the neighborhood of item *v*, $y_{ue}$ denotes the actual label of user *u* for item *e*, and $\tilde{\psi }_{r_{v,e}}^{u}$ is the weighted relationship between item and its neighbor *e*. This formula calculates the smoothed label $\hat{y}_{UV}^{\textrm{LS}}$ for item *v* by taking the weighted average of the neighboring items’ labels.

To optimize the model, a cross-entropy loss function $L_{loss}$ with a label smoothing regularization term is used. Cross-entropy loss quantifies the difference between the predicted and actual probability distributions. The full loss function is expressed as follows:

17 $$\begin{aligned} L_{\textrm{loss}}=L_{\textrm{basic}}+\rho L_{\textrm{ls}}+\lambda \parallel F\parallel _{2}^{2}, \end{aligned}$$

Here, $L_{basic}$ is the basic cross-entropy loss, used to calculate the error between the actual click label $y_{uv}$ and the predicted click probability $\hat{y}_{uv}$, and is expressed as:

18 $$\begin{aligned} L_{\textrm{basic}}=\sum _{(u,v)\in Y}-(y_{uv}\log (\sigma (U^{\textrm{T}}V)))+(1-y_{uv})\log (1-\sigma (U^{\textrm{T}}V), \end{aligned}$$

On top of this, the label smoothing regularization term $L_{ls}$ uses the smoothed neighbor labels to constrain the model, improving its ability to predict unannotated items. $\rho$ is the weight coefficient for the label smoothing term, controlling its impact on the total loss. Finally, $\lambda \Vert F\Vert _{2}^{2}$ is the L2 regularization term, used to prevent overfitting, where $\lambda$ is the corresponding weight.

By computing the click probability using the dot product of the user interest vector *U* and the item feature vector *V*, and combining this with the cross-entropy loss function with a label smoothing regularization term, the model effectively optimizes prediction accuracy.

## Experiments

### Datasets

To better evaluate the proposed algorithm in this paper, we used a user-item interaction dataset enhanced with a knowledge graph. The experiment utilized two widely-used datasets: the Book-Crossing^28^ and MovieLens-1M^29^ datasets. The corresponding knowledge graph was constructed by Microsoft’s Satori. Table 1 provides detailed information about the two datasets.

**Table 1:** Data details of the dataset.

Dataset	Book	Movie
User Quantity	19636	3254
Item Quantity	20013	1856
Interactive Quantity	177265	522365
Entity Quantity	25754	7355
Relation Quanity	18	5
Triple Quanity	60025	15255

### Experimental setup

To verify the effectiveness and performance of the proposed DNGAKG model, several representative recommendation algorithms were selected as baselines for comparison, including MKR, RippleNet, SPGCN, and LightGCN.

MKR utilizes deep neural networks to model the interactions between items and entities in the knowledge graph, with the cross-compression unit serving as its core component to enhance item representations. RippleNet adopts a memory-network-based framework that propagates user preferences through multi-hop paths in the knowledge graph to capture personalized interests. SPGCN improves traditional graph convolutional networks by optimizing nonlinear activation and feature transformation modules to enhance aggregation efficiency. LightGCN is a vanilla collaborative filtering model that relies solely on user–item interaction data and does not utilize any side information.

All experiments were conducted on a Windows 10 system with Python 3.8, using TensorFlow 2.5.0, scikit-learn 0.24.1, and NumPy 1.20.1. The hardware environment consisted of an Intel Core i9-13700HX CPU, an NVIDIA GeForce RTX 4070 GPU, and 32 GB of RAM. Model parameters were initialized using the Xavier initialization strategy, and the Adam optimizer was employed for training.

For data preparation, each dataset was randomly split into training, validation, and test sets with a ratio of 7:2:1. Following common practice in implicit feedback recommendation, negative samples were generated by randomly selecting items that a user had not interacted with. Each positive interaction was paired with an equal number of negative samples. All models were trained for a fixed number of epochs, and the model with the best performance on the validation set was selected for final evaluation on the test set.

The hyperparameter settings for the proposed model are summarized in Table 2. Key parameters include the number of sampled neighbors *N*, the number of hops *H*, and the embedding dimension *d*. The hyperparameters of baseline models were set according to the configurations reported in their original papers. When necessary, parameters were further tuned on the validation set to ensure fair comparison.

**Table 2:** Hyperparameter settings.

Super parameter	Book	Movie
$N-\mu$	35	36
$N-i$	8	5
$H-\mu$	3	2
$H-i$	4	2
*d*	32	16
$\gamma$	0.1	0.1
$\rho$	0.5	1
$\lambda$	$2\times 10^{-5}$	$1\times 10^{-7}$
$\eta$	$2\times 10^{-5}$	$2\times 10^{-2}$
Batch Size	512	256

### Evaluation metrics

The evaluation of the recommendation algorithms focused on two scenarios: click-through rate (CTR) prediction and Top-K recommendation. For CTR prediction, the performance was assessed using AUC and F1-score metrics.

AUC is a measure of how well the model differentiates between classes, with values ranging from 0 1, where higher scores indicate better performance.

F1-score, on the other hand, is a metric that balances precision and recall, calculated using the following formula:

19 $$\begin{aligned} F_{1}=\frac{2\cdot P\cdot R}{P+R}, \end{aligned}$$

where *P* is precision, and represents recall. For the Top-K recommendation scenario, recall was used as the evaluation metric, which reflects whether the recommendation system successfully captures the user’s potential interests in items.

## Results

In this experiment, we used two widely used datasets, Book-Crossing and MovieLens-1M, to evaluate the performance of the recommendation algorithm. These two datasets help us further verify the effect of the model in complex scenarios by integrating knowledge graphs. In the experiment, our model TNRA is used as the main model to compare with multiple existing recommendation algorithm models, including MKR, RippleNet, SPGCN and LIGHTGCN. These models have different characteristics. For example, MKR uses deep neural networks to infer the interaction between items and entities in the knowledge graph, RippleNet automatically discovers the potential connection path between user history and recommended items, SPGCN optimizes nonlinear activation and feature transformation modules, and LIGHTGCN introduces lightweight GCNs to alleviate data sparsity.

As shown in Table 3, the TNRA model outperformed other models on both datasets. On the Book-Crossing dataset, TNRA achieved an AUC of 0.763 and an F1 score of 0.688, while on the MovieLens-1M dataset, it attained an AUC of 0.983 and an F1 score of 0.951. The TNRA model’s AUC and F1 scores were 0.72% to 1.62% higher than those of the comparison models, demonstrating a notable performance advantage.

**Table 3:** Results of AUC and F1 in CTR prediction.

Model	Book		Movie	
	AUC	F1	AUC	F1
MKR^30^	0.733	0.615	0.912	0.836
Social-RippleNet^31^	0.721	0.675	0.958	0.857
SPGCN^32^	0.738	0.662	0.965	0.928
LightGCN^33^	0.752	0.679	0.976	0.936
TNRA	0.763	0.688	0.983	0.951

The results demonstrate that the TNRA model outperforms other models in recommendation tasks. This superior performance is primarily attributed to TNRA’s dual neighbor information aggregation mechanism, which operates on both the user and item sides. On the user side, TNRA employs a graph attention mechanism to effectively capture users’ historical interests and combines this with diffusion paths in the knowledge graph to generate potential interest vectors. On the item side, TNRA utilizes graph convolution to capture the local neighborhood structure, further optimizing the loss function with label regularization. These enhancements allow the model to better identify users’ potential preferences and item feature associations, resulting in more accurate recommendations.

**Fig. 4:**
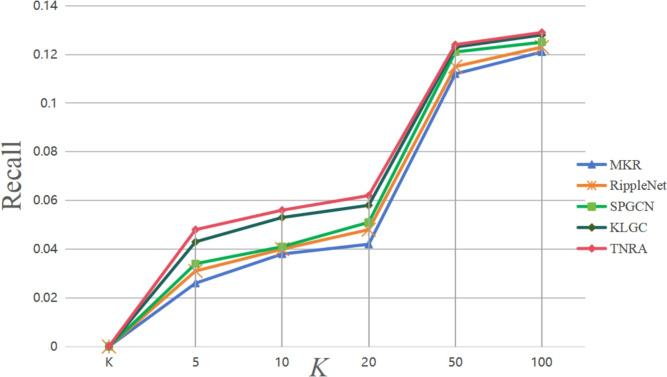
Recall of Top-K recommendations on the Book-Crossing dataset.

**Fig. 5:**
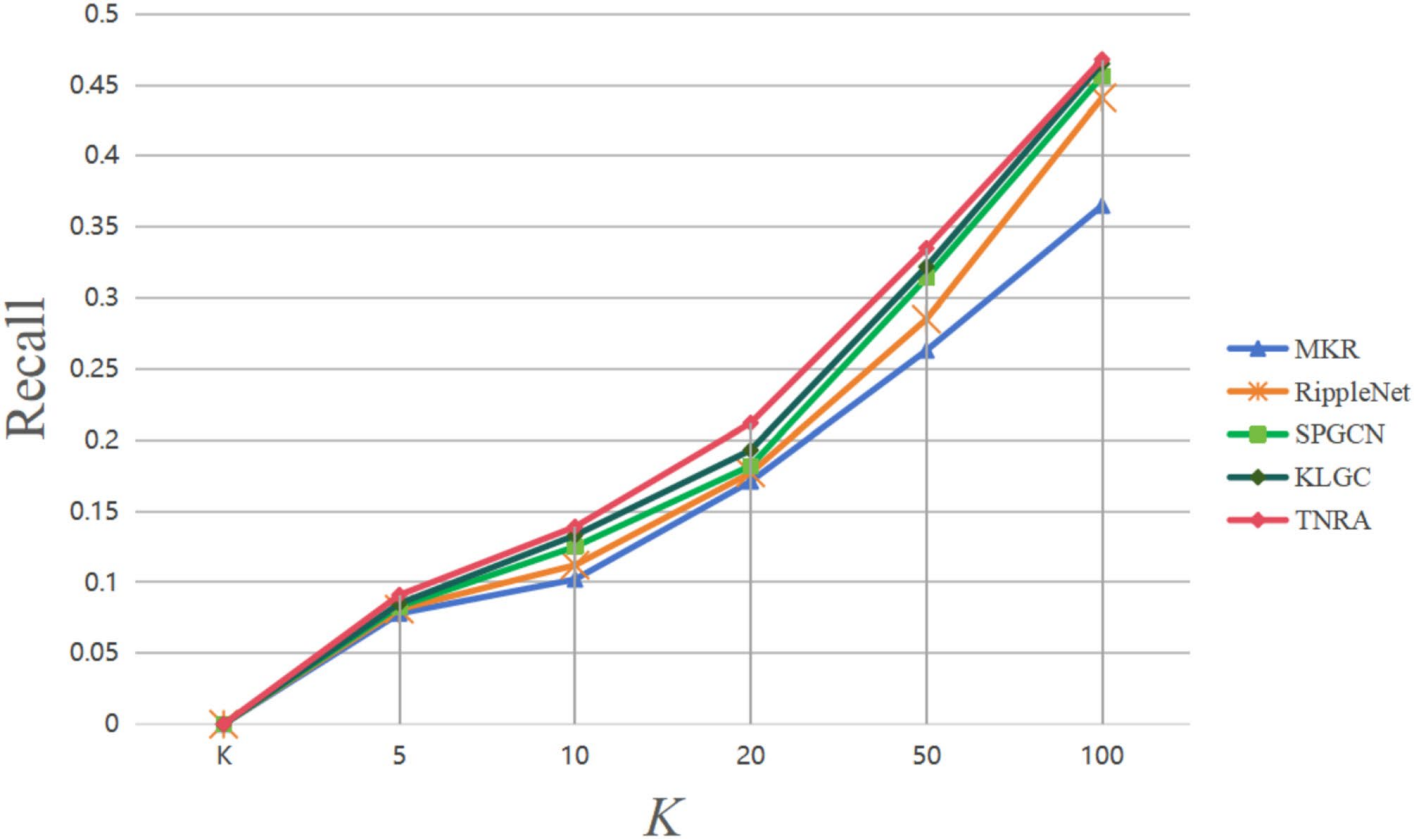
Recall rate of Top-K recommendations on the MovieLens-1M dataset.

For the Top-K recommendation scenario, recall is evaluated as the key metric, where different K values (5, 10, 20, 50, 100) were tested to observe changes in recall performance. The experimental results are depicted in Figs. 4 and 5. Using two widely adopted dataset’s Book-Crossing and MovieLens-1M the TNRA model was compared with other well-known algorithms like Social-RippleNet, SPGCN, and LightGCN to assess recall performance in Top-K recommendations. Recall measures the proportion of relevant items out of the K items recommended to a user, with a higher recall indicating the model’s ability to more effectively capture users’ interests.

**Table 4:** Comparison of training efficiency and resource consumption.

Model	Time per Epoch (s)	GPU Memory (GB)	Parameters (M)
MKR	18.4	3.1	12.6
Social-RippleNet	21.7	3.4	14.2
SPGCN	19.9	3.2	11.8
LightGCN	15.6	2.7	9.4
TNRA	22.8	3.6	15.1

Table 4 reports the training efficiency and resource consumption of different models. Although TNRA introduces additional computational cost due to multi-hop neighborhood expansion and attention-based aggregation, its training time per epoch remains comparable to other graph-based methods such as Social-RippleNet and SPGCN.

The moderate increase in GPU memory usage mainly comes from maintaining multi-hop ripple sets and attention parameters. Nevertheless, the overall resource consumption of TNRA remains within a practical range, and the performance improvements demonstrated in Table 3 justify the additional computational overhead.

The experimental results for the Book-Crossing dataset, as shown in Fig. 4, indicate that as the K value increases, the recall of all models steadily improves. However, the TNRA model consistently outperforms the others at every K value. Specifically, at K=100, TNRA achieves a recall rate of 0.129, compared to 0.128 for LIGHTGCN, 0.125 for SPGCN, and 0.123 for Social-RippleNet. This demonstrates that TNRA’s dual neighbor aggregation mechanism more effectively captures users’ potential preferences and improves recommendation accuracy.

Similarly, the results for the MovieLens-1M dataset in Fig. 5 further confirm the TNRA model’s superiority. At K=100, TNRA reaches a recall rate of 0.468, outperforming LIGHTGCN (0.465), SPGCN (0.456), and Social-RippleNet (0.441). Even at K=20, TNRA shows a higher recall rate of 0.335 compared to LIGHTGCN’s 0.322, SPGCN’s 0.314, and Social-RippleNet’s 0.285, reinforcing its effectiveness in capturing user preferences and delivering more accurate recommendations.

The TNRA model’s strong recall performance on both datasets can be attributed to several key factors. First, on the user side, TNRA employs a graph attention mechanism that begins with users’ historical interest items and progressively diffuses potential interest information, capturing the complex interactions between users and items. Second, on the item side, TNRA leverages a graph convolutional network to aggregate information from neighboring nodes, producing more accurate and representative item feature vectors. Additionally, label regularization is incorporated to reduce overfitting. Lastly, the model calculates the inner product of the user and item feature vectors to predict the likelihood of user engagement, further improving the accuracy of recommendations.

In the experiment, the hyperparameters are adjusted to study the impact of each hyperparameter value on the recommendation performance of the article model. Table 5 shows the impact of different hop counts H, item-side neighbor sampling number N and vector dimension d on the evaluation index AUC value.

**Table 5:** The impact of hop count H, item-side neighbor sampling number N, and vector dimension d on the AUC value.

H	Book	Movie	N	Book	Movie	d	Book	Movie
1	0.722	0.935	2	0.725	0.974	4	0.736	0.945
2	0.752	0.988	4	0.752	0.988	8	0.758	0.973
3	0.763	0.975	8	0.768	0.975	16	0.744	0.983
4	0.756	0.963	16	0.732	0.977	32	0.755	0.972

In this experiment, we assessed the AUC values of the model using two widely recognized recommendation system datasets, Book-Crossing and MovieLens-1M. AUC (Area Under the Curve) is a key metric for evaluating a model?s predictive accuracy, as it measures the area under the ROC curve, which reflects how well the model distinguishes between positive and negative samples. AUC values range from 0 to 1, with higher values indicating better classification performance.

During the experiment, several important hyperparameters of the TNRA model were adjusted, including the number of hops, the number of neighbors sampled on the item side (N), and the embedding vector dimension (d). Changes in these hyperparameters directly influence the model’s ability to represent data and its overall predictive accuracy. The results, as detailed in Table 5, illustrate the effect of varying these parameters on the AUC scores under different configurations.

In the experiment on the number of hops H, the AUC values on both datasets increased at first and then decreased as the number of hops increased. On the Book-Crossing dataset, when H = 3, the AUC reached the highest value of 0.763, while on the MovieLens-1M dataset, the AUC reached 0.988 when H = 2. This indicates that in the knowledge graph diffusion process, a smaller number of hops effectively captures relevant information from neighboring nodes, but as the number of hops increases, noise information accumulates, leading to a performance decline. The results suggest that in practical applications, one-hop or two-hop neighbors in the knowledge graph are usually more relevant, while too many hops may introduce irrelevant information, negatively affecting the model’s predictive performance.

In the experiment on the number of sampled neighbors N, the AUC values also exhibited a similar trend of increasing first and then decreasing. On the Book-Crossing dataset, when N = 8, the AUC was the highest at 0.768, while on the MovieLens-1M dataset, N = 4 yielded the highest AUC of 0.988. This demonstrates that a reasonable number of sampled neighbors is critical for capturing item features, where an appropriate amount of neighbors provides valuable information, but too many sampled neighbors introduce noise, reducing model performance. Therefore, the number of sampled neighbors on the item side needs to be controlled within an optimal range to ensure the model can efficiently aggregate valuable node information.

In the experiment on vector dimension d, the results showed that as the embedding vector dimension increased, the AUC values also improved within a certain range. On the Book-Crossing dataset, when d = 32, the AUC reached 0.755, while on the MovieLens-1M dataset, d = 16 achieved an AUC of 0.983. This indicates that an appropriate vector dimension helps the model better capture the features of users and items and effectively encode the information in the graph. However, excessively large vector dimensions may introduce redundant information, extending training time without further improving model performance.

The TNRA model demonstrates excellent performance in recommendation tasks by aggregating bidirectional neighbor information on both the user and item sides and incorporating a graph attention mechanism and label smoothing regularization. TNRA fully leverages user’s historical interests and item’s neighboring node information. Through the graph convolutional network, it efficiently aggregates this information to generate high-quality user and item embedding vectors, and the dot product of these vectors calculates the user’s click probability on items. The experimental results show that TNRA has a significant advantage in AUC over other methods, making it particularly suitable for complex recommendation scenarios and sparse data environments.

We also clarify the use of textual information in our experiments: the compared baselines do not take review text as input in our implementation, whereas TNRA incorporates text embeddings as auxiliary semantic features to enrich node representations. Therefore, to isolate the contribution of text features and other components, we conduct an ablation study as follows.

We evaluate three TNRA variants: (1) *TNRA w/o Text*, which removes the text embeddings and uses only graph-based embeddings as the initial representations; (2) *TNRA w/o Label Smoothing*, which sets $\rho =0$ in Eq. (17) and optimizes only the basic cross-entropy loss with L2 regularization; and (3) *TNRA w/o Attention*, which replaces the attention coefficients $\alpha$ in Eq. (4) with uniform weights, i.e., $\alpha _{(h,r,t)}^{k}=1/|S_u^{k}|$, resulting in an unweighted mean aggregation over ripple triples. The ablation results on both datasets (AUC/F1 and Recall@K) are reported in Table 6.

**Table 6:** Ablation study on TNRA components.

Variant	Book		Movie	
	AUC	F1	AUC	F1
TNRA (full)	0.763	0.688	0.983	0.951
TNRA w/o Text	0.754	0.679	0.978	0.944
TNRA w/o Label Smoothing	0.758	0.683	0.980	0.947
TNRA w/o Attention	0.756	0.681	0.979	0.946

The ablation results show that removing text embeddings consistently degrades performance, indicating that textual semantics effectively complement sparse implicit feedback. Disabling label smoothing leads to a performance drop, verifying its regularization effect for better generalization. Replacing attention with uniform aggregation also reduces AUC/F1, demonstrating that attention helps the model focus on more informative triples during multi-hop preference propagation.

## Conclusion

This study addresses the challenge of improving recommendation accuracy in personalized systems, particularly in situations with data sparsity and cold start problems. To tackle this, a personalized recommendation algorithm that integrates GAT with large-scale text features is proposed. The approach leverages users’ historical interaction data and knowledge graphs to dynamically aggregate multi-hop neighbor information between users and items. By utilizing GCN, it captures the neighborhood topology of items, producing more accurate user and item feature vectors. The TNRA model was compared against well-established algorithms like MKR, Social-RippleNet, and SPGCN. Results indicated that TNRA achieves consistent improvements over these models in terms of AUC and F1 scores, particularly excelling in sparse data environments. In the Top-K recommendation tasks, TNRA consistently demonstrated higher recall rates across various K values, confirming its superiority in capturing users’ potential interests.

Beyond accuracy, we further analyze the computational overhead and practical constraints. As reported in Table 4, TNRA requires 22.8 s per epoch and 3.6 GB GPU memory, which is comparable to other graph-based baselines (e.g., Social-RippleNet: 21.7 s, 3.4 GB; SPGCN: 19.9 s, 3.2 GB), while introducing a moderate parameter increase (15.1 M). This overhead mainly comes from multi-hop ripple set expansion and attention-based aggregation, but remains within a practical training budget in our experiments.

Regarding scalability to industrial-scale datasets, the main bottlenecks are (i) multi-hop neighborhood expansion and (ii) message passing over large knowledge graphs. In practice, scalability can be improved via neighbor sampling (already adopted in our item-side aggregation), limiting hop number *H*, and offline precomputation/caching of high-frequency neighborhood representations. These strategies can reduce the effective per-request graph computation and make the model more suitable for large-scale deployment.

For real-time recommendation, a feasible deployment paradigm is to separate offline and online stages: offline training and periodic embedding refresh are performed on distributed infrastructure, while the online stage retrieves precomputed user/item embeddings and computes $\hat{y}_{uv}$ with lightweight dot products and small MLP modules. For cold-start or rapidly changing users, online updates can be approximated by aggregating a small set of recent interactions with shallow hops (e.g., $H\le 2$) and bounded neighbor sampling to meet latency constraints.

We also highlight potential risks in graph-based propagation. First, excessive message passing may lead to over-smoothing, where node representations become indistinguishable; this motivates using a small hop number and residual/skip aggregation across hops. Second, knowledge graph noise and spurious relations may introduce irrelevant signals; therefore, robust attention weighting, relation pruning, and noise-aware sampling are important directions to further improve reliability.

In summary, TNRA improves recommendation performance under sparse interactions while maintaining manageable computational cost. Future work will focus on further reducing training/inference overhead, enhancing scalability on larger graphs, and incorporating explicit noise-control and anti-over-smoothing mechanisms for more stable real-world deployment.

## Data Availability

The data that support the findings of this study are available on request from the corresponding author. The data are not publicly available due to privacy or ethical restrictions.
